# Dual Covalent Cross-Linking Networks in Polynorbornene: Comparison of Shape Memory Performance

**DOI:** 10.3390/ma14123249

**Published:** 2021-06-12

**Authors:** Haotian Zhao, Qinghong Zhang, Xinlong Wen, Gongliang Wang, Xiaowen Gong, Xinyan Shi

**Affiliations:** Key Laboratory of Rubber-Plastics, Ministry of Education, College of Polymer Science and Engineering, Qingdao University of Science and Technology, Qingdao 266045, China; 2019020037@mails.qust.edu.cn (H.Z.); 4020020059@mails.qust.edu.cn (Q.Z.); 2020020028@mails.qust.edu.cn (X.W.); 2020020026@mails.qust.edu.cn (G.W.); 2018020009@mails.qust.edu.cn (X.G.)

**Keywords:** polynorbornene, dual covalent cross-linking networks, shape memory performance

## Abstract

In this work, tetrakis(dimethyllamino)ethylene (TDAE) plasticized polynorbornene (PNB) was used as the matrix, sulfur (S) and dicumyl peroxide (DCP) were simultaneously used as crosslinking agents to construct dual covalent cross-linking networks in PNB. The effects of different amounts of cross-linkers on the crosslinking degree, mechanical property, glass transition temperature, and PNB shape memory performance were investigated. Two crosslinking mechanisms were examined by Fourier transform infrared spectrometer and Raman spectrometer. The results showed that sulfur-rich cross-linked PNB exhibited a higher crosslinking degree, tensile strength, and slightly higher glass transition temperature than the DCP-rich system. Cross-linked PNB presented better shape memory performance than the uncross-linked one. Sulfur-rich cross-linked PNB showed even better shape memory behavior than the DCP-rich system, both with a shape fixation ratio of over 99% and a shape recovery ratio of over 90%. The reaction mechanism of sulfur and DCP in cross-linking PNB was different. Sulfur reacted with the α-H in PNB to form monosulfide bonds, disulfide bonds, and polysulfide bonds in PNB and the number of polysulfide bonds increased with increased amounts of sulfur. DCP reacted with the double bonds in PNB to form C-C covalent bond crosslinking networks. The crosslinking mechanism revealed that the sulfur-containing cross-linked bonds, especially polysulfide bonds, were more flexible and bore large deformation, which gave the PNB excellent mechanical properties and ensured a higher shape entropy elastic recovery ratio.

## 1. Introduction

Shape memory polymer (SMP) is a kind of intelligent material, which can change its original shape and store a temporary shape. SMP can recover to its original shape by external stimuli (such as temperature, light, electricity, heat, and magnetic and chemical reagents) [1,2,3,4,5]. In 1951, Chang and Read [6] first observed the shape memory effect in AuCd alloy materials, and proposed the concept of shape memory. Since then, the discovery of cross-linked polyethylene had completely opened the door of SMP exploration [7]. SMPs have the advantages of being light weight and having an adjustable shape, high elasticity, and high-cost performance. It has broad application prospects in the fields of biology, chemistry, military, microelectronics, medicine and health [8,9,10]. Especially in the context of the increasingly tense international situation and the global new crown epidemic, the development of shape memory polymers is crucial.

Among them, the thermoplastic shape memory polymer (TSMP) is a relatively mature and easy-to-control one. It only needs to be placed above the transition temperature (T_trans_) to cause deformation, and “freeze” to deform after cooling. The temporary shape is obtained, and the temperature is finally restored. However, due to the need to repeatedly change the temperature before and after deformation, which consumes extra time and energy, people have proposed the concept of reversible plasticity shape memory polymer (RPSMP) [11,12]. RPSMP is different from the common TSMP in achieving the deformation and fixation of the material through the reorganization of the chain segment structure below T_trans_ and the constant temperature after forced movement in the glass state [12,13,14]. However, the T_trans_ of RPSMP should not be too low, it is best to be located near room temperature, which is conducive to the deformation of the material at room temperature. It can avoid the tedious temperature change process, saving time and energy, and is beneficial to the deformation of large-size or complex structure products. Lin [15] prepared SMPs with reversible plastic shape memory properties by introducing hindered phenolic small molecule additives AO-80 into zinc acrylate (ZDA) vulcanized epoxidized natural rubber (ENR). The composite material can achieve plastic deformation with a strain of up to 300% below T_g_, and maintain the shape after the external force. When the shape is restored, it can generate a higher recovery stress than traditional SMPs. Therefore, it has a high driving force drive and high grip force.

The following is the research conducted by our research team in the early stages of reversible plastic shape memory polymer (RPSMP). Qu [16] selected polynorbornene rubber (PNR, containing plasticizing oil and carbon black) and polynorbornene resin (PNB) as a matrix material, prepared oil-extended PNR, PNR/EPDM blends, low-oil-extended PNB (Lo-PNB) and PNB composites, and explored the shape memory properties of PNB composites; Zhang [17] used PNB as the matrix to explore the molecular chain, temperature, time, and other factors that affect the shape memory performance of materials, and the shape memory performance of PNB was optimized by blending. Previous studies showed that PNB is a typical reversible plastic shape memory polymer. First, its ultra-high molecular weight above 3.0 × 10^6^ can provide conditions for shape fixation. Second, the glass transition temperature of PNB is around 37.3 °C, which can be deformed at room temperature. Based on the above characteristics, PNB has obvious advantages in the field of RPSMP. However, PNB must be combined with plasticizing oil to change its processing performance, and needs to meet the requirements of low energy consumption. The shape fixation ration still has room for improvement. Therefore, it is necessary to systematically understand its influencing factors and deformation process. As is known, the cross-linked network has a very prominent influence on polymer properties. Therefore, it is particularly critical to explore the influence of the cross-linking network on PNB shape memory performance. Xiao [18] introduced dicumyl peroxide (DCP) in PNB to build a cross-linking network in the PNB matrix, which improved the shape recovery ratio of PNB. Gong [19] prepared polynorbornene/zinc dimethacrylate (ZDMA)/dicumyl peroxide (DCP) composites and explored the effect of a double covalent cross-linking network on the shape memory performance of PNB. It was also found that the double cross-linked network stores more energy during deformation, providing a higher ratio of shape recovery. In view of the previous study, in this work tetrakis(dimethyllamino)ethylene (TDAE) plasticized polynorbornene (PNB) is used as the matrix, sulfur (S) and dicumyl peroxide (DCP) are simultaneously used as crosslinking agents to construct dual covalent cross-linking networks in PNB. The effects of different amounts of cross-linkers on the crosslinking degree, mechanical property, glass transition temperature, and PNB shape memory performance were investigated. Two crosslinking mechanisms were examined by Fourier transform infrared spectrometer and Raman spectrometer as well. This study is expected to provide more reference for improving shape memory performance of PNB.

## 2. Experimental

### 2.1. Materials

Polynorbornene (PNB) with average molecular weight of 3 × 10^6^ was purchased from STARTECH Co., Austria; Tetrakis(dimethyllamino)ethylene (TDAE, Grade REPSOL TDAE 1996) with density of 0.950 kg/m^3^ and viscosity of 16~21 cSt was obtained from Pingxiang Shenglaite Chemical Technology Co., Pingxiang, China. Sulfur (S); and DCP-40 (DCP effective content is 40%, 60% is calcium carbonate carrier) was purchased from Henan Yixiang Chemical Co., China.

### 2.2. Preparation of the Samples

Firstly, PNB and TDAE (PNB: TDAE = 80:20) were introduced in a Haake Torque Rheometer (Haake Rheocord90, Germany) and blended at 70 rpm for 7 min at a temperature of 70 °C. Secondly, sulfur and DCP were added to the mix for 6 min according to the formula in Table 1. Finally, the above compound was placed on a two-roll mill with a temperature of 50 °C and a roll pitch of 1 mm, and mixed to obtain the samples with a thickness of 1 mm. A flat plate molding machine (XLB, Yadong Machinery, Qingdao, China) was used to preheat at 170 °C for 1 min, and then maintain pressure at 10 MPa for 3 min. Then, a cold press at 10 MPa and room temperature for 6 min was carried out to obtain samples of 1 mm thick.

### 2.3. Crosslinking Characteristic

Tested by Moving Die Rheometer MDR2000 (ALPHA, Livonia, MI, USA), with a temperature of 170 °C for 40 min.

### 2.4. Glass Transition Temperature

Tested by DSC204F1 differential scanning calorimeter (Netzsch, Selb, Germany); scanning temperature of 50~150 °C, and the temperature rise and fall ratio of 10 °C/min.

### 2.5. Physical and Mechanical Properties

Tested by Z005 universal electronic tensile tester (Zwick Company, Ulm, Germany), with a crosshead speed of 50 mm/min.

### 2.6. Dynamic Mechanical Properties

Tested by DMA242 thermal analyzer produced (Netzsch, Germany), cut into a spline with a size of about 20 × 5 × 2 mm, double cantilever mode, frequency 1 Hz, under an air atmosphere, with a scanning temperature of −50~150 °C, and a temperature rise and fall ratio of 3 °C/min.

### 2.7. Fourier Transform Infrared Spectrometer (FTIR)

Tested by VERTEX70 type Fourier transform infrared spectrometer (BRUKER, Mannheim, Germany), ATR mode.

### 2.8. Raman Spectrometer

The specimen was attached to the glass slide. And the measurements were performed at room temperature using a Raman spectrometer (DM 2500 M Ren) with a 532 nm Nd: YAG laser excitation source.

### 2.9. Shape Memory Performance

Tested by DMA-Q800 (TA Company, New Castle, DE, USA), cut into a spline with a size of about 20 × 5 × 2 mm. Firstly, the temperature was raised to 30 °C above the T_g_ of the PNB sample, and stabilized for 5 min. The length of the sample was recorded as ε_p_. We applied 50% strain to each sample, and the length of the sample was marked as ε_load_. Then, the temperature was lowered to 30 °C below the T_g_. The external force was removed, and the temperature was stabilized for 5 min, and the length of the sample was marked as ε. Finally, the temperature was raised from T_g_−0 °C to T_g_+30 °C, with a heating ratio of 5 °C/min, and the shape recovery process of the sample was completed by maintaining a constant temperature for 30 min. Finally, the length of the sample was marked as ε_rec_. 

The shape fixing ratio (R_f_) and shape recovery ratio (R_r_) were calculated as follows:

R_f_ = (ε − ε_p_)/(ε_load_ − ε_p_) × 100% (1)

R_r_ = (ε_load_ − ε_rec_)/(ε_load_ − ε_p_) × 100% (2)

## 3. Results and Discussions

### 3.1. Crosslinking Characteristics

**Figure 1:**
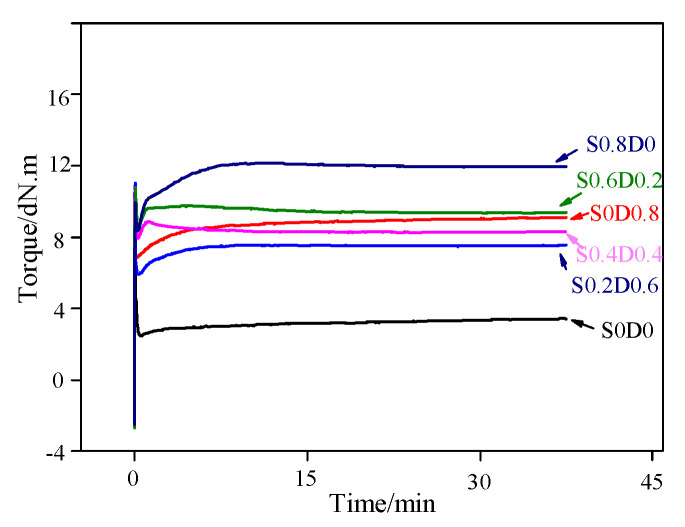
Cross-linking curves of PNB.

Figure 1 shows the cross-linking curves of PNB tested by MDR2000. It can be seen from Figure 1 that the torque of S0D0 increased with the extension of testing time, which is caused by the entropic elasticity of the macromolecular structure of the PNB. It is well known that M_H_–M_L_ reflected the crosslinking density of cured polymer. It can be seen in Table 2 that the M_H_–M_L_ of PNB of S0.8D0 was the highest and that PNB of S0D0.8 was second highest. The reason for this was that the molar mass of sulfur (32 g/mol) is much less than that of DCP (270 g/mol); the number of free radicals from sulfur is much larger than that from the same weight of DCP. Consequently, with the decrease in sulfur content, the crosslinking network density and t_90_ of the samples gradually decreased. When the ratio of sulfur to DCP was 1:1, both the crosslinking network density and t_90_ had the minimum value. This might be because the free radicals produced by sulfur and DCP could combine with each other and terminate, reducing the number of free radicals reacting with the matrix.

### 3.2. Glass Transition Temperature

**Figure 2:**
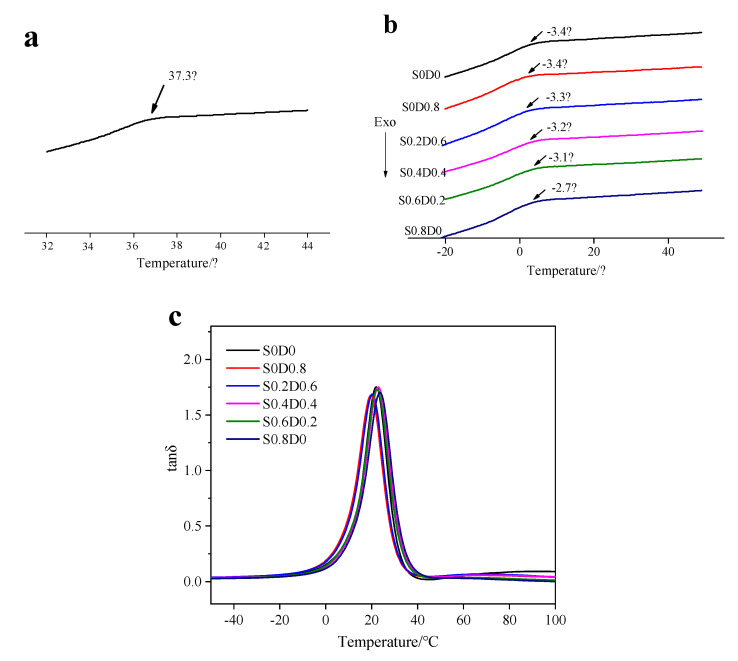
The glass transition temperature of PNB: (**a**) PNB; (**b**) PNB with sulfur and DCP by DSC; (**c**) PNB with sulfur and DCP by DMA).

Figure 2a shows the T_g_ of pure PNB at 37.3 °C. It can be seen from Figure 2b that the glass transition temperature of PNB (S0D0) decreased to −3 °C with 20 phr TDAE. The plasticizer oil could immerse into the PNB, weaken the interaction between molecular chains, and enhance the movement ability of chain segments. T_g_ for the other PNB samples did not change significantly, especially the one with only DCP (S0D0.8). However, T_g_ for the PNB with only sulfur (S0.8D0) slightly shifted to a higher temperature (from −3.4 °C to −2.7 °C), which is attributed to the higher crosslinking density restricting the chain movement of PNB. The glass transition temperature increased with the addition of sulfur. It further proved that the same amount of sulfur in PNB obtained a higher crosslinking degree than that of DCP. From the loss factor diagram of the material in Figure 2c, it can be seen that all samples had a tanδ peak, the T_g_ peak (due to the different DMA test modes, the measured T_g_ value was different from the DSC, and the DSC test result shall prevail).

### 3.3. Mechanical Properties

**Figure 3:**
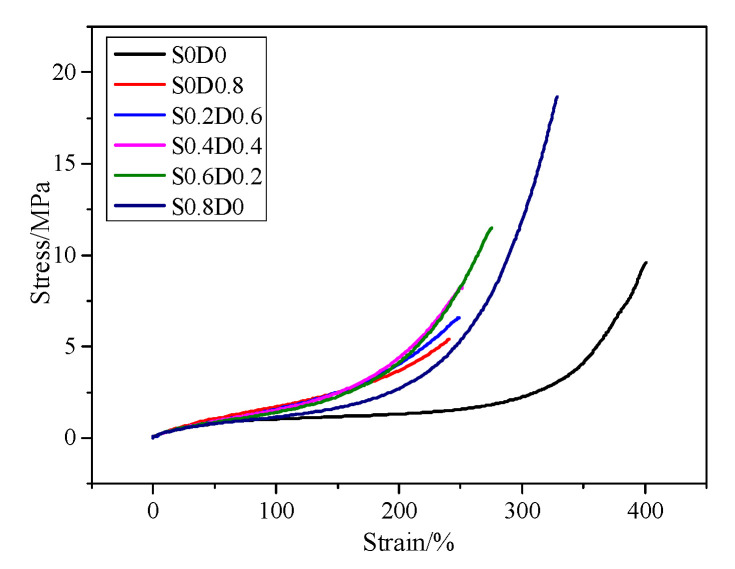
The stress-strain curves of PNB at 24 °C.

**Figure 4:**
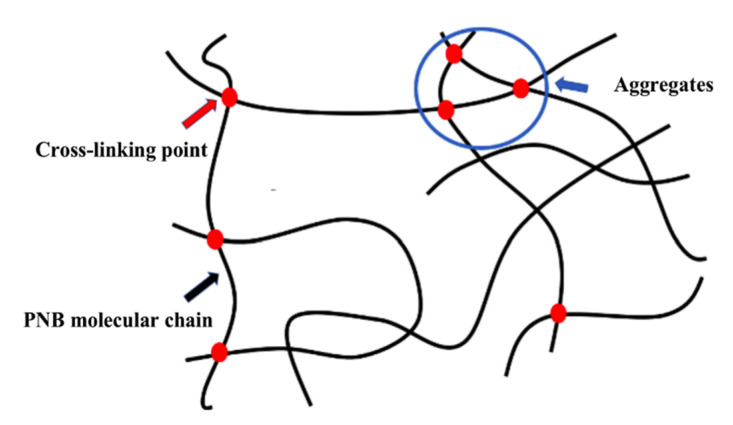
Schematic diagram of the structural characteristics with DCP crosslinking network.

Figure 3 shows the tensile curves for PNB samples. Table 3 shows the mechanical properties of PNB at 24 °C. It can be seen from Figure 3 that the curves of S0D0 without the crosslinking network were similar to elastomers (a sharp upward trend at higher elongation). Because PNB had an ultra-high molecular weight and a long single molecular chain, the molecular chains of PNB with plasticizer were easy to move during the tensile test; when the orientation of chains occurred, the stress increased suddenly. After adding sulfur and DCP, the crosslinking network in PNB restricted the movement of the molecular chains and the stress of PNB increased in advance. Moreover, the mechanical properties of PNB with sulfur (S0.8D0) were better than DCP (S0D0.8); both tensile strength and elongation at break increased greatly [20]. The chemical properties of different crosslinked bond networks are different. The tensile strength of specimens increased with a decrease in bond energy, which is related to the bond length. The energy of C-C bonds (351.7 kJ/mol) is higher than that of polysulfide bonds (267.9 kJ/mol) and the length of C-C bonds (1.53 × 10^2^ pm) is shorter than that of polysulfide bonds (1.90~2.07 × 10^2^ pm) [21,22,23]. The stress distribution was not uniform during the network deforming; the short bonds (C-C bonds) suffered stress first [24]. The molecular chains of PNB had no time to orient, causing a bad mechanical property. Compared with C-C bonds, the single and multi-sulfur bond crosslinking networks in PNB had lower bond energy, which could break earlier to prevent stress concentration. Therefore, with the increase in sulfur, the mechanical properties of PNB gradually improved. It can be seen from Table 2 that the 100% tensile stress of PNB decreased with the increase in sulfur. This is due to the fact that crosslinking networks of DCP with lower bond length mainly suffered stress at 100% tensile stress. Figure 4 shows the schematic diagram of the structural characteristics with DCP crosslinking network.

### 3.4. Dynamic Mechanical Performance Analysis

**Figure 5:**
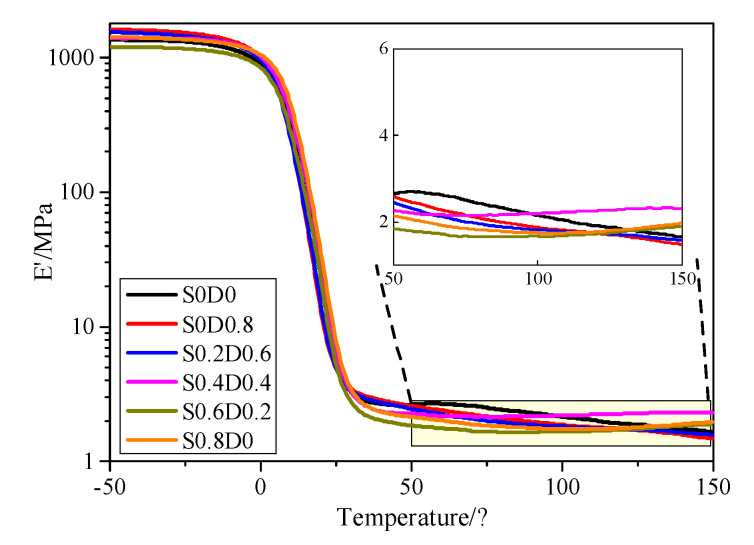
Dynamic mechanical properties of PNB.

In order to evaluate the shape memory effect for the crosslinked PNB, a DMA test was carried out as shown in Figure 5. It can be seen from Figure 5 that the curves of different PNB were basically the same. PNB in a glass state had a high storage modulus at low temperature. The storage modulus decreased rapidly when the temperature increased over the transition temperature of PNB. There were two platforms on the curves (low and high temperature platforms). PNB was in glass state with higher strength and the modulus at a low temperature platform. At a high temperature platform, PNB was in a high elastic state with a lower storage modulus. In the range of 0–25 °C, the glass transition of PNB occurred and the modulus increased sharply from low to high temperature. It can be seen from Table 4 that the change of E’ had at least two orders of magnitude before and after glass transition, which was a necessary condition for the material to have shape memory properties [25]. High E’ at low temperature means that the segments of reversible phase are frozen during the cooling process and the molecular chains do not have time to relax. PNB could store more energy with higher stress. Low E’ at high temperature means that PNB could release more energy. Therefore, it can provide more energy for shape recovery and the recovery ratio would be faster.

The influence of two networks on the properties of PNB was further analyzed for the high elastic state above 50 °C. The high modulus of elasticity of PNB with more sulfur was almost unchanged with the increase in temperature. It indicated that highly crosslinked PNB segments were bound by covalent networks. The E’ of PNB with 0.6 and 0.8 phr sulfur increased slightly around 125 °C. The PNB segments were bound by the network of sulfur; the molecular chains reflected the entropic elasticity recovery trend. In addition, a small number of polysulfide bonds broke and rearranged at high temperature, forming monosulfide and disulfide bonds. The bond length became shorter and PNB became hard. A C-C crosslinking network with a lower crosslinking degree was formed in PNB with higher DCP content. The segments of PNB were disentangled and the center of the molecular chains was shifted at high temperature, causing the modulus to decrease slightly [26,27,28].

### 3.5. Shape Memory Performance

The PNB used in this work were mild crosslinked samples. Under slow tension, the samples always had a thermodynamic equilibrium conformation, which belongs to reversible deformation or equilibrium deformation. According to the first law of thermodynamics, the change of internal energy *dU* is as follows:

*dU* = *dQ* − *dW* (3)

where, *dQ* is the heat exchange between PNB and the outside environment. According to the second law of thermodynamics, a constant temperature reversible process is as follows:

*dQ* = *TdS* (4)

where, *S* is the entropy of PNB and *dW* is the exchange between PNB and the outside environment, including the expansion of volume change and the elongation of tensile deformation.

*dW* = *pdV* − *Fdl* (5)

*dU* = *Tds* − *pdV* + *Fdl* (6)

It is assumed that the volume of PNB remains unchanged during the tensile process (*pdV* = 0).

*dU* = *Tds* + *Fdl* (7)

(∂*U*/∂*l*)_*T*,*V*_ = *T* (∂*S*/∂*l*)_*T*,*V*_ + *F* (8)

*F* = (∂*U*/∂*l*)_*T*,*V*_ − *T* (∂*S*/∂*l*)_*T*,*V*_ (9)

The Formula (9) shows that the internal energy of PNB changes slowly in the tensile process [29]. It is assumed that there are no intramolecular and intermolecular forces in PNB, and the internal energy of the system remains unchanged when the elastomer deforms.

*F* = −*T* (∂*S*/∂*l*)_*T*,*V*_ (10)

It shows that the entropic elasticity of the system in the tensile process contributes to the elastic recovery force of PNB (entropic elasticity). The molecular chains of PNB are random and curly in the free state. When tensile deformation occurs, the molecular chains are forced to change from a curly state to a stretched state; the order degree increases while the conformational entropy decreases. Due to the existence of molecular thermal motion, the molecular chains have the tendency to spontaneously recover to the original curly state, resulting in the elastic restoring force. The recovery trend of conformational entropy is more intense due to the increase in conformational complexity [30]. 

In the initial free state, due to the existence of thermal motion, the network chains between the crosslinking points can be regarded as random coil chains whose terminal distance distribution conforms to Gauss distribution. The PNB has a large conformation entropy. Under external force, the whole network was deformed. The results showed that the order degree of the network chains increased while the conformational entropy decreased when all the chains deformed.

*σ* = *n*_1_*kT*(*λ*^−1^/*λ*^2^) = *ρRT*/*Mc*(*λ*^−1^/*λ*^2^) (11)

The Formula (11) is the equation of the state of the crosslinked elastomer. Where *n_1_* represents the number of chains per unit volume, *ρ* is the crosslinking density of crosslinked elastomers, *Mc* is the average molecular weight of the network chains, *λ* is the material tensile ratio, and *R* is the molar constant of gas.

The Formula (11) shows that the elastic stress is directly proportional to the temperature when high elastic deformation occurs. The elastic stress increases with the increase in temperature. The stress is inversely proportional to the average molecular weight of the network chains. The ability of shape recovery increases with the increase in crosslinking density.

**Figure 6:**
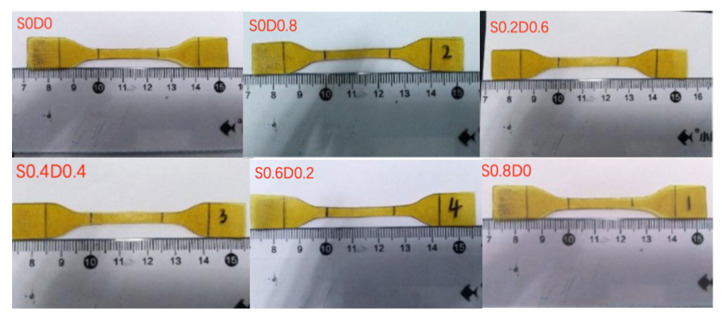
Shape memory test samples of PNB.

**Figure 7:**
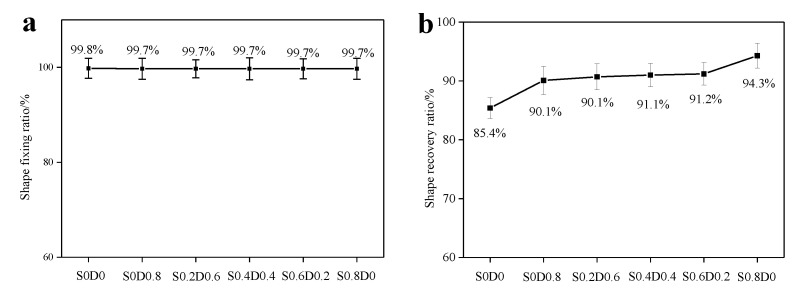
The shape fixation ratio (**a**) and shape recovery ratio (**b**) of PNB (Tg−0 °C–Tg+30 °C).

Figure 6 shows shape memory test samples of PNB. The shape memory properties of PNB were tested by dynamic thermomechanical analysis (DMA). The PNB was cooled to T_g_−30 °C for fixation with the control of 50% strain. Then, the temperature was increased to T_g_+30 °C for recovery. The formula was used to calculate the fixation ratio and recovery ratio (Figure 7). As can be seen from Figure 7a, all of the PNB materials had an excellent fixation ratio (about 99.7%). This showed that PNB had only a small elastic deformation recovery after removing the force; PNB could basically maintain the original temporary shape. When the temperature was above the glass transition temperature of PNB, the movement ability of the chain segments was enhanced. The molecular chains gradually returned to the original state under the effect of entropic elasticity after removing the force. The shape recovery ratio of PNB increased significantly with the addition of the crosslinking agent. This was because the crosslinking network in PNB increased resilience and shape recovery ratio. With the increase in sulfur, the density of sulfur crosslinking network was higher, which provides stronger resilience for shape recovery.

**Figure 8:**
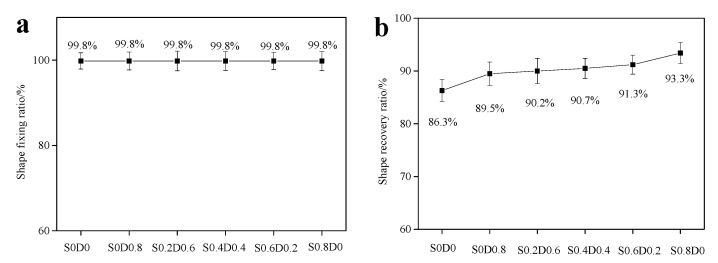
The shape fixation ratio (**a**) and shape recovery ratio (**b**) of PNB (T_g_−15 °C–T_g_+30 °C).

In order to further characterize the shape memory properties, the PNB were fixed at Tg−15 °C and recovered at Tg+30 °C, which is shown in Figure 8. It was found that the PNB could still maintain great fixation ratio and recovery ratio after increasing the fixed temperature through calculation. The crosslinking network made the PNB molecular chains fix by the crosslinking network, ensuring excellent shape memory properties.

### 3.6. Mechanism Analysis of Crosslinking Reaction

**Figure 9:**
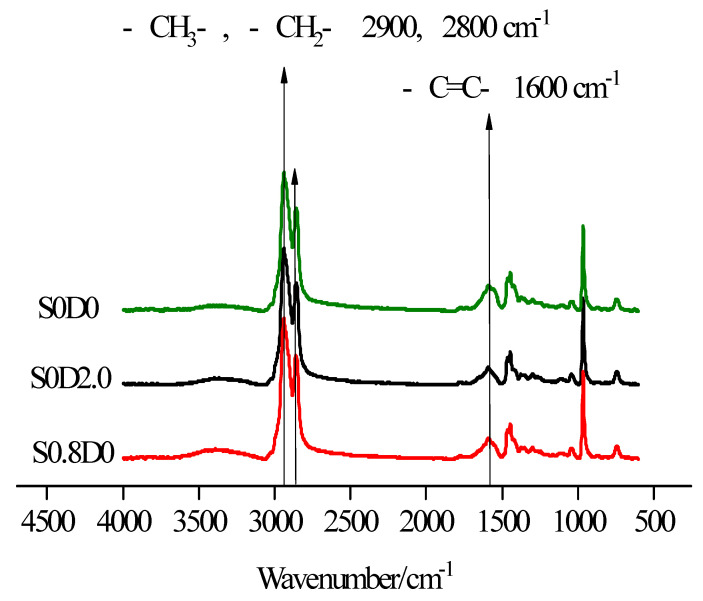
Infrared spectrum analysis of PNB.

The S0D0, S0D0.8 and S0.8D0 were tested by FTIR, as shown in Figure 9.

Table 5 shows ratio of infrared absorption peak area. The infrared absorption peak of C=C appeared at 1600 cm^−1^ for all three samples. We compared the area of C=C infrared absorption peak at 1600 cm^−1^ with –CH_2_ at 2900 cm^−1^ and the results. The ratio of S0D0 to S0.8D0 was almost the same, which indicated that the addition of sulfur could not cause the change of the number of double bonds in PNB. It indicated that the active points of the sulfur crosslinking reaction in PNB were not double bonds. However, the ratio of S0D2.0 was lower, which indicated that the number of double bonds in PNB decreased with the addition of DCP. The results showed that the reaction activity of DCP crosslinked with PNB was a double bond, while the sulfur crosslinking was not a double bond.

**Figure 10:**
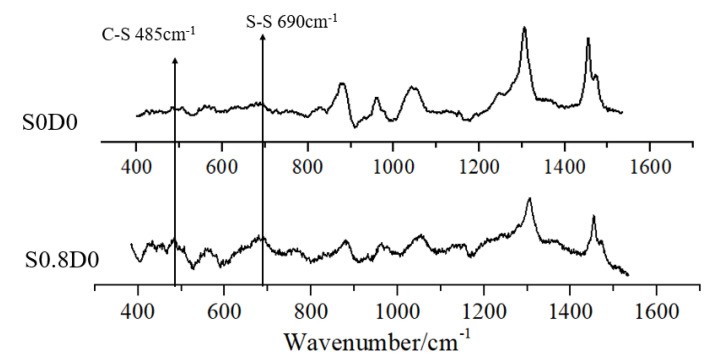
Raman spectrum analysis of PNB.

The Raman spectrum of PNB was analyzed in order to further characterize the type of crosslinking bonds in sulfur crosslinked with PNB (Figure 10). There were absorption peaks of -C-S- at 485 cm^−1^ and -S-S- at 690 cm^−1^ in S0.8D0, respectively. Theoretically, there is one -C-S- bond and no -S-S- bond in the monosulfide bond of -C-S-C-, so -S-S-:-C-S- should be 0:2. There is one -S-S- bond and two -C-S- bonds in the disulfide bond of -C-S-S-C-, so the ratio of -S-S-:-C-S- should be 1:2. If polysulfide bonds of -C-Sx-C exist, then the ratio would be more than 1:2, as shown in Table 6.

The real absorption peak area ratio at 690 cm^−1^ vs. 485 cm^−1^ by Raman spectrometer is shown in Table 7. It can be seen that the ratio in sulfur-rich samples was greater than 1:2. This indicated that there were monosulfide bonds, disulfide bonds, and polysulfide bonds in the sulfur-rich cross-linked PNB. With the increase in sulfur, the ratio further increased, indicating that the number of polysulfide bonds increased.

**Figure 11:**
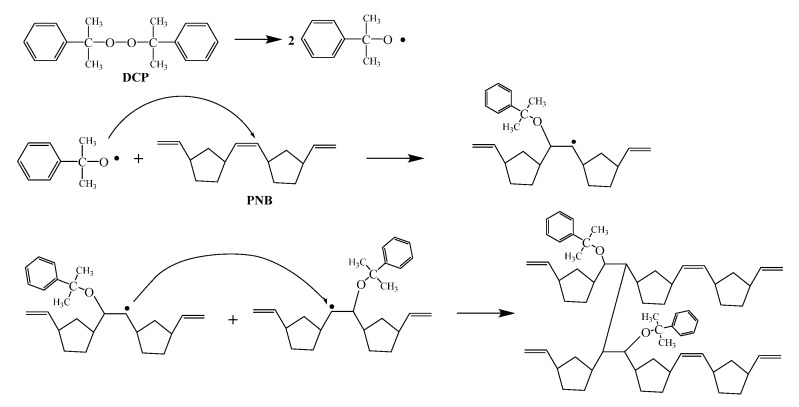
Reaction mechanism of DCP crosslinking PNB.

**Figure 12:**
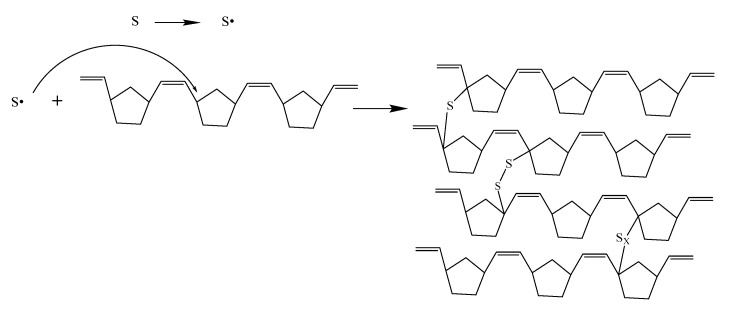
Reaction mechanism of sulfur crosslinking PNB.

**Figure 13:**
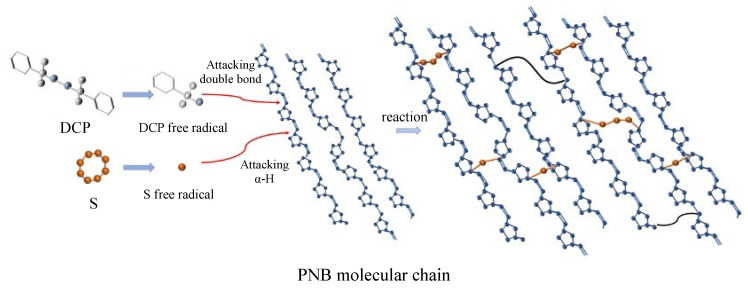
Schematic diagram of two crosslinked networks in PNB.

The reaction mechanism is shown in Figure 11 and Figure 12. The DCP decomposed to generate free radicals, causing the double bonds on the main chains of PNB to open and form new free radicals. The addition of the reaction of free radicals to the adjacent molecular chains was carried out to realize the cross-linking, forming a polymer which is similar to the spherical crosslinking network. The sulfur radical reacted with α-H in PNB to form single sulfur bonds and multi sulfur bonds after the addition of sulfur (Figure 13).

## 4. Conclusions

(1) Sulfur and DCP belong to two different types of cross-linking agents, and the reaction mechanism of cross-linking PNB is different. Through infrared and Raman spectroscopy analysis of the composite material, it was found that sulfur reacts with the α-H on the PNB double bond to produce sulfur-containing crosslinks DCP and PNB double bonds undergo crosslinking reactions to produce C-C covalent bonds.
(2) There are differences in bond length and bond energy between sulfur-containing cross-linked bonds and carbon–carbon cross-linked bonds, making PNB materials of different cross-linking types perform differently in various properties. The carbon–carbon cross-linked bond produced after DCP cross-linking has a short bond length and a large bond energy, and adjacent double bonds form an aggregate structure after the addition of polymerization, which adversely affects the mechanical properties of the DCP cross-linked PNB composite material.
(3) The sulfur-containing cross-linked bond has a long bond length, a small bond energy, and a strong ability to bear external forces under large strains. This gives the sulfur cross-linked PNB material excellent mechanical properties and also ensures its entropic elasticity deformation in the shape memory test. It has sufficient restoring force during recovery, high shape recovery ratio, and the material has the best shape memory performance.

## Figures and Tables

**Table 1:** PNB formula with different amounts of cross-linkers.

Samples	S0D0	S0D0.8	S0.2D0.6	S0.4D0.4	S0.6D0.2	S0.8D0
PNB	80.0	80.0	80.0	80.0	80.0	80.0
TDAE	20.0	20.0	20.0	20.0	20.0	20.0
S	0	0	0.2	0.4	0.6	0.8
DCP	0	0.8	0.6	0.4	0.2	0.0

Note: Amounts of all materials are presented in phr. The sample was named SXDY, where X represented the phr of S and Y represented the phr of DCP; TDAE was used as a plasticizer to improve the processing properties of PNB.

**Table 2:** Cross-linking characteristics of PNB.

Samples	S0D0	S0D0.8	S0.2D0.6	S0.4D0.4	S0.6D0.2	S0.8D0
t_90_/min	31.33	11.30	9.95	4.01	6.57	7.50
M_L_/dN.m	2.22	6.73	5.96	7.94	8.08	8.18
M_H_/dN.m	2.72	8.75	7.62	8.87	9.78	13.28
M_H_−M_L_/dN.m	0.50	2.02	1.56	0.93	1.70	5.10

**Table 3:** Mechanical properties of PNB at 24 °C.

Samples	S0D0	S0D0.8	S0.2D0.6	S0.4D0.4	S0.6D0.2	S0.8D0
Tensile strength/MPa	9.61 ± 0.97	5.48 ± 2.35	6.57 ± 0.43	8.31 ± 2.15	11.50 ± 0.95	18.67 ± 1.47
Elongation at break/%	400.7 ± 12.5	240.4 ± 24.6	249.0 ± 14.8	251.1 ± 36.9	275.1 ± 11.2	328.1 ± 19.3
Modulus at 100%/MPa	1.02 ± 0.03	1.71 ± 0.84	1.57 ± 0.10	1.53 ± 0.24	1.40 ± 0.11	1.14 ± 0.11
Modulus at 200%/MPa	1.32 ± 0.05	3.70 ± 1.70	4.04 ± 0.48	4.40 ± 1.13	4.13 ± 0.67	2.71 ± 0.66

**Table 4:** Storage modulus and ratio of PNB @T_g_+30 °C.

Samples	E’T_g_−30 °C/MPa	E’T_g_+30 °C/MPa	E’T_g_−0 °C/E’T_g_+30 °C
S0D0	1336.9	4.2	317.9
S0D0.8	1577.9	4.1	389.1
S0.2D0.6	1462.3	4.0	364.3
S0.4D0.4	1393.2	4.6	366.3
S0.6D0.2	1186.8	3.7	320.8
S0.8D0	1394.2	4.7	300.46

**Table 5:** Ratio of infrared absorption peak area.

S0D0	S0D0.8	S0.8D0
0.41	0.37	0.40

**Table 6:** The theoretical peak area ratio at 690 cm^−1^ (-S-S-) vs. 485 cm^−1^ (-C-S-).

-C-S-C-	-C-S-S-C-	-C-Sx-C
0:2	1:2	>1:2

**Table 7:** Raman absorption peak area ratio.

S0.2D0.6	S0.4D0.4	S0.6D0.2	S0.8D0
0.43	0.75	1.21	1.64

## Data Availability

The data presented in this study are available on reasonable request from the corresponding author. The data are not publicly available due to the data being further processed for other purposes.

## References

[1] Meng H., Li G. (2013). A review of stimuli-responsive shape memory polymer composites. Polymer.

[2] Wu T., O’Kelly K., Chen B. (2014). Poly(vinyl alcohol) particle-reinforced elastomer composites with water-active shape-memory effects. Eur. Polym. J..

[3] Chan B., Low Z., Heng S., Chan S.Y., Owh C., Loh X.J. (2016). Recent Advances in Shape Memory Soft Materials for Biomedical Applications. ACS Appl. Mater. Interfaces.

[4] Cohades A., Branfoot C., Rae S., Bond I., Michaud V. (2018). Progress in Self-Healing Fiber-Reinforced Polymer Composites. Adv. Mater. Interfaces.

[5] Kanu N.J., Gupta E., Vates U.K., Singh G.K. (2019). Self-healing composites: A state-of-the-art review. Compos. Part A Appl. Sci. Manuf..

[6] Song G., Ma N., Li H.N. (2006). Applications of shape memory alloys in civil structures. Eng. Struct..

[7] Li F.K., Zhu W., Zhang X., Zhao C.T., Xu M. (1998). Shape memory effect of slightly-crosslinked polyethylene. Chin. J. Polym. Sci..

[8] Yue L., Liu F., Mekala S., Patel A., Gross R.A., Manas-Zloczower I. (2019). High Performance Biobased Epoxy Nanocomposite Reinforced with a Bacterial Cellulose Nanofiber Network. ACS Sustain. Chem. Eng..

[9] Rybak A., Jarosinski L., Gaska K., Kapusta C. (2018). Graphene nanoplatelet-silica hybrid epoxy composites as electrical insulation with enhanced thermal conductivity. Polym. Compos..

[10] Liu Y., Du H., Liu L., Leng J. (2014). Shape memory polymers and their composites in aerospace applications: A review. Smart Mater. Struct..

[11] Ortega A.M., Yakacki C.M., Dixon S.A., Likos R., Greenberg A.R., Gall K. (2012). Effect of crosslinking and long-term storage on the shape-memory behavior of (meth)acrylate-based shape-memory polymers. Soft Matter.

[12] Rodriguez E.D., Luo X., Mather P.T. (2011). Linear/Network Poly(ε-caprolactone) Blends Exhibiting Shape Memory Assisted Self-Healing (SMASH). ACS Appl. Mater. Interfaces.

[13] Xiao R., Choi J., Lakhera N., Yakacki Cr M., Frick C.P., Nguyen T.D. (2013). Modeling the glass transition of amorphous networks for shape-memory behavior. J. Mech. Phys. Solids.

[14] Tao X. (2011). Recent advances in polymer shape memory. Polymer.

[15] Lin T., Tang Z., Guo B. (2014). New Design Strategy for Reversible Plasticity Shape Memory Polymers with Deformable Glassy Aggregates. ACS Appl. Mater. Interfaces.

[16] Qu M. (2017). Study on Shape Memory Properties of Poly (Norbornene). Ph.D. Thesis.

[17] Zhang M.L., Ji X.X., Shi X.Y. (2019). Study on key influencing factors of reversible plastic shape memory of poly (norbornene). Acta Polym. Sin..

[18] Xiao Y. (2019). Construction of Polynorbornene Crosslinking Network and Its Shape Memory Effect. Ph.D. Thesis.

[19] Gong X., Yin H., Zhang M., Shi X. (2020). Effects of dual-crosslinking networks on shape memory performance of polynorbornene. J. Appl. Polym. Sci..

[20] Lendlein A., Zotzmann J., Feng Y., Alteheld A., Kelch S. (2009). Controlling the switching temperature of biodegradable, amorphous, shape-memory poly(rac-lactide)urethane networks by incorporation of different comonomers. Biomacromolecules.

[21] Meng H., Li G. (2013). Reversible switching transitions of stimuli-responsive shape changing polymers. J. Mater. Chem. A.

[22] Leng J., Wu X., Liu Y. (2010). Infrared light—Active shape memory polymer filled with nanocarbon particles. J. Appl. Polym. Sci..

[23] Wu X., Chen G.Y., Zhang W., Liu X., Xu H. (2017). A Plant-Transpiration-Process-Inspired Strategy for Highly Efficient Solar Evaporation. Adv. Sustain. Syst..

[24] Wischke C., Schossig M., Lendlein A. (2014). Shape-Memory Effect of Micro-/Nanoparticles from Thermoplastic Multiblock Copoly-mers. Small.

[25] Huang W.M., Lee C.W., Teo H.P. (2006). Thermomechanical Behavior of a Polyurethane Shape Memory Polymer Foam. J. Intell. Mater. Syst. Struct..

[26] He Y., Cao Y., Wang Y. (2018). Progress on photothermal conversion in the second NIR window based on conjugated polymers. Asian J. Org. Chem..

[27] Mitra S., Chattopadhyay S., Bhowmick A.K. (2010). Effects of quasi-nanogel particles on the rheological and mechanical properties of natural rubber: A new insight. J. Appl. Polym. Sci..

[28] Hel C.L., Bounor-Legaré V., Catherin M., Lucas A., Thèvenon A., Cassagnau P. (2020). TPV: A New Insight on the Rubber Morphology and Mechanic/Elastic Properties. Polymers.

[29] Bhattacharya A.B., Raju A.T., Chatterjee T., Naskar K. (2020). Development and characterizations of ultra-high molecular weight EPDM/PP based TPV nanocomposites for automotive applications. Polym. Compos..

[30] Leng J., Lan X., Liu Y., Du S. (2011). Shape-memory polymers and their composites: Stimulus methods and applications. Prog. Mater. Sci..

